# Digitalised primary care in the UK: a qualitative study of the experiences of minoritised ethnic communities

**DOI:** 10.3399/BJGP.2024.0308

**Published:** 2024-10-29

**Authors:** Farjana Islam, Sara Bailey, Gina Netto

**Affiliations:** The Urban Institute, Heriot-Watt University, Edinburgh.; Institute of Educational Technology, the Open University.; The Urban Institute, Heriot-Watt University, Edinburgh, UK.

**Keywords:** digital access to health care, digitalisation of primary care, ethnic and racial minorities, intersectionality, minoritised ethnic, primary health care, qualitative research, racialised barriers

## Abstract

**Background:**

Barriers to accessing and using primary care services among minoritised ethnic communities have been extensively evidenced in the UK. However, the impact of the rapid digitalisation of these services on these communities remains under-researched.

**Aim:**

To explore the impact of digitalisation on access to and use of primary care services among minoritised ethnic communities.

**Design and setting:**

Underpinned by a critical realist intersectional approach, and employing qualitative research methods, this study explores minoritised ethnic individuals’ experiences of digital primary care in the UK.

**Method:**

In total, 100 minoritised ethnic adults who identify as Black African, Black Caribbean, Bangladeshi, Indian, Pakistani, Chinese, and of mixed or multiple ethnic heritage in four sites in the UK were purposively recruited and interviewed. Interviews were thematically analysed to increase understanding of how individuals’ ethnicity intersects with other characteristics (for example, language, age, gender, socioeconomic status) to identify constraints and enablements to accessing health care.

**Results:**

Minoritised ethnic individuals’ access to digital primary care is impeded by factors such as digital precarity (for example, inadequate devices, internet connectivity, and digital literacy skills), a lack of language support, and staff shortcomings in responding to ethnically diverse populations. Intergenerational support and bespoke offerings by general practices in some areas enable some individuals to overcome some of the constraints.

**Conclusion:**

The rapid digitalisation of primary care services is replicating and potentially exacerbating barriers to using these services among minoritised ethnic communities, a finding that merits urgent attention by practitioners and policymakers.

## Introduction

The performance of a healthcare system must be assessed, at least in part, by the extent of equitable access to and use of primary care services.^1^^,^^2^ In the UK, however, persistent racialised inequalities in access and use are evident.^3^^,^^4^ For example, according to the English GP Patient Survey of 2023, Bangladeshi, Pakistani, and other Asian groups have a less positive experience of booking GP appointments than other ethnic groups.^4^^–^6 Such disparities compel us to pay greater attention to the equitability of GP services, which is the focus of this article. This is especially the case at times of significant transformation such as the changes that are entailed by the rapid digitalisation of GP services.

Underpinned by the ‘digital first’ primary care agenda, as outlined both in the 2019 *NHS Long Term Plan* (England) and the 2018 *Digital Health and Care Strategy* (Scotland), primary care patients are increasingly being steered towards using digital channels to access services. In this context, concerns have been raised that racialised inequalities may be exacerbated. The Topol Review,^7^ for example, highlights the risk of digitalisation leading to increased racialised exclusions because minoritised ethnic communities often lack the resources and capacity to engage with digital services.^5^^,^^8^^–^10 By minoritised ethnic communities, we mean communities who identify as Black African, Black Caribbean, Bangladeshi, Pakistani, Indian, Chinese, and of mixed or multiple ethnic heritage. These communities have been minoritised by their ethnicity and racial or cultural features (for example, skin colour, language) and are vulnerable to multiple forms of discrimination.^11^^–^15 Given the significant role of ethnicity as a social determinant of health,^10^^,^^16^^,^^17^ and recognition that ethnicity has remained a neglected parameter in efforts to ensure equitable health outcomes, this observation raises considerable concerns.

The aims of this article are two-fold. First, it aims to raise awareness of the complex challenges some individuals from minoritised ethnic communities experience in accessing and using digital health platforms. These challenges, when combined with pre-existing inequalities in accessing health services,^1^^–^4^,^^18^^–^20 may hinder timely access to health care or even exclude some individuals altogether. Second, the article aims to inform the future delivery of primary care services by increasing awareness of areas where the equitability of GP services needs to be enhanced. By adopting a critical realist intersectional approach,^12^^,^^21^ we extend understanding beyond the relationship between ethnicity as a single dimension of identity and use of digital health services. This approach enables us to examine how individuals’ ethnicity interacts with other dimensions of inequality such as age, gender, religious background, income, and geographical location, to influence use of these services. Based on Archer,^22^^,^^23^ we identify ethnicity-induced ‘constraints’ and ‘enablements’ in the home and in general practices, which act either singly or in combination to influence access to digital primary care services.

**Table:** How this fits in

The extant literature demonstrates that minoritised ethnic communities’ access to and use of primary care services has long been impeded by factors such as a lack of linguistic and cultural sensitivity among primary care practitioners, resulting in lower levels of satisfaction among these groups. However, the impact of the recent digitalisation of primary care services on these communities, a significant policy and practice development, has yet to be adequately researched. Underpinned by a critical realist approach, which recognises that structural systems and processes at the national level and in general practices are strongly influenced by societal power dynamics, and interact with the material and social resources of individuals in the home, this article explores the experiences of minoritised ethnic communities through an intersectional lens. It identifies clusters of ‘constraints’ and ‘enablements’ related to digital access at the general practice and household or individual levels. It demonstrates that the digitalisation of primary care is replicating, and in some cases even exacerbating, pre-existing barriers in accessing GP services, a finding that merits urgent consideration by policymakers and practitioners.

## Method

We selected qualitative methods to reveal the complexity of the processes that influence minoritised communities’ exclusion from digital engagement in England and Scotland. The four case study sites — Manchester, Bradford, and the London Borough of Tower Hamlets in England, and Glasgow in Scotland — were purposively chosen because they have a significant population of these communities. According to the English 2021 Census, 43.2%, 38.9%, and 60.8% of the population from Manchester, Bradford, and the London Borough of Tower Hamlets, respectively, are of Asian, Black, and mixed or multiple ethnic heritage. Glasgow was selected because, according to the Scottish 2011 Census, it has the highest representation of non-White minoritised ethnic communities in Scotland (11.58%). Fieldwork in these sites generated nuanced insights into the ways in which ethnicity interacts with factors at the household and general-practice level to facilitate or constrain minoritised ethnic communities’ digital access.

### Theoretical framework

Underpinned by a critical realist approach,^21^^–^27 which recognises that structural systems and processes at the national and general practice levels are strongly influenced by societal power dynamics^28^ and interact with the resources of individuals in the home,^21^ we explored the experiences of minoritised ethnic communities through an intersectional lens. National-level systems and processes include macro-level imperatives, such as NHS England and NHS Scotland’s digital strategies, while at the level of general practices, initiatives to encourage the use of online services impact directly on access to primary care services.

The intersectional framework employed views minoritised ethnic individuals as members of multiple groups rooted in embodied and inseparable categories of social difference. Such an approach enables examination of the interplay of multiple dimensions of inequality to highlight issues that give rise to ethnicity-related disparities and exclusions.^21^^,^^29^ The critical realist approach to intersectionality theory enables the identification of ‘constraints’ and ‘enablements’ that interact with multiple dimensions of identity and inequality to respectively hinder and facilitate access to and use of health services.^22^^,^^23^^,^^29^

### Data collection and analysis

We carried out in-depth one-to-one interviews with 100 adult minoritised ethnic individuals either in-person or online between August and December 2022. The interviewees were purposively recruited with community partners’ support to ensure that the ethnic groups that are most at risk of digital exclusion owing to their disadvantaged socioeconomic status,^13^ such as Bangladeshis, Pakistanis, and Black Africans, were well represented. Twenty per cent of interviewees were older (aged >65 years) and 60% were female.

Four researchers, including the first two authors, conducted the interviews, most of which were 1-hour long. Data collection processes at all stages adhered to the ethical protocols of the universities involved and General Data Protection Regulation (GDPR) 2018 guidelines. With the help of a consent form, researchers explained how data would be used, that information provided would be kept confidential, and that participants could withdraw from the research at any point. Participants were offered £20 gift-voucher incentives.

We observed variation in participants’ language proficiency and confidence levels in speaking, reading, writing, and understanding English. For instance, some stressed that they were proficient in spoken but not written English, while others reported that they could read but not speak English. Considerable diversity was also observed in terms of digital engagement, ranging from high levels of competency to non-use of the internet.

Smartphones were identified as the most frequently used digital device. A few individuals had access to computers or laptops, although the latter were often shared with others. Table 1 summarises the demographic characteristics of participants such as age, gender, ethnicity, level of education, and employment status.

**Table 1:** Demographic profile of 100 interview participants

**Participants’ sociodemographic attributes**	**Percentage**	
**Age, years**	Young (18–35)	23
	Middle (36–65)	57
	Older (>65)	20

**Self-described gender**	Male	39
	Female	60
	Queer	1

**Self-described ethnicity**	Bangladeshi	23
	Black African	21
	Pakistani	17
	Indian	13
	Black Caribbean	12
	Chinese	9
	Dual Heritage	2
	Other Black and Asian	3

**Level of education**	Postgraduate	15
	Graduate	21
	College	23
	High school	23
	Primary school or lower	9
	Other or not stated	9

**Employment status**	Employed	38
	Unemployed	24
	Retired	22
	Student	5
	Self-employed	3
	Others	8

A semi-structured interview schedule was developed to explore minoritised communities’ use of online services, particularly GP services, and their experiences of online harm with a view to recommending policy initiatives for inclusive and anti-racist ‘digital first’ primary care. Interview questions explored the following issues: access to digital devices and the internet; digital literacy; access to support; experiences of using online GP services; and perceptions about the use of such services.

Eighty-six interviews were conducted in English and 14 in the participants’ first language, of which seven interviews were conducted by the first author in Bengali and the rest with the support of professional interpreters. All interviews were audio-recorded except for 12 where consent was only given to take fieldnotes. Audio-recorded interviews were professionally transcribed, involving the use of secure websites to ensure confidentiality and data security. Where audio-recordings were not available, fieldnotes were structured according to the interview guide and coded in NVivo (version 12) alongside the interview transcripts. All information and transcripts were stored in a secure Dropbox folder and anonymised.

The interviews generated a large dataset of subjective narratives of participants’ experiences in accessing and using online GP services, which revealed the influence of their membership of multiple social and disadvantaged groups (for example, age and gender) and status with respect to various dimensions of inequality (proficiency in English, digital poverty, digital literacy). The third author conceptualised the development of a novel critical realist intersectional lens to interpret the data through an inductive and reflexive thematic analysis.^30^^–^34 The first two authors familiarised themselves with the dataset for an in-depth understanding of participants’ lived realities within the ‘localised’ socio-structural landscape of digital primary care services in England and Scotland, and generated codes that led to the construction of themes and sub-themes. These themes (for example, access to digital services, language barriers) and sub-themes (for example, digital connectivity, intergenerational support) reflect the complexity of diverse participants’ engagement with online GP services. All three authors then considered how intersecting aspects of participants’ identities and their position with respect to various dimensions of inequality impacted on access to and use of digital services. All authors identified the ways in which ‘constraints’ and ‘enablements’ both within the household and within GP surgeries interacted with each other. All three authors identify as female racialised minorities working in higher education in the UK, and are aware of their privileged position with respect to power dynamics in the research process and knowledge of health services when compared with the majority of participants.

## Results

**Figure 1:**
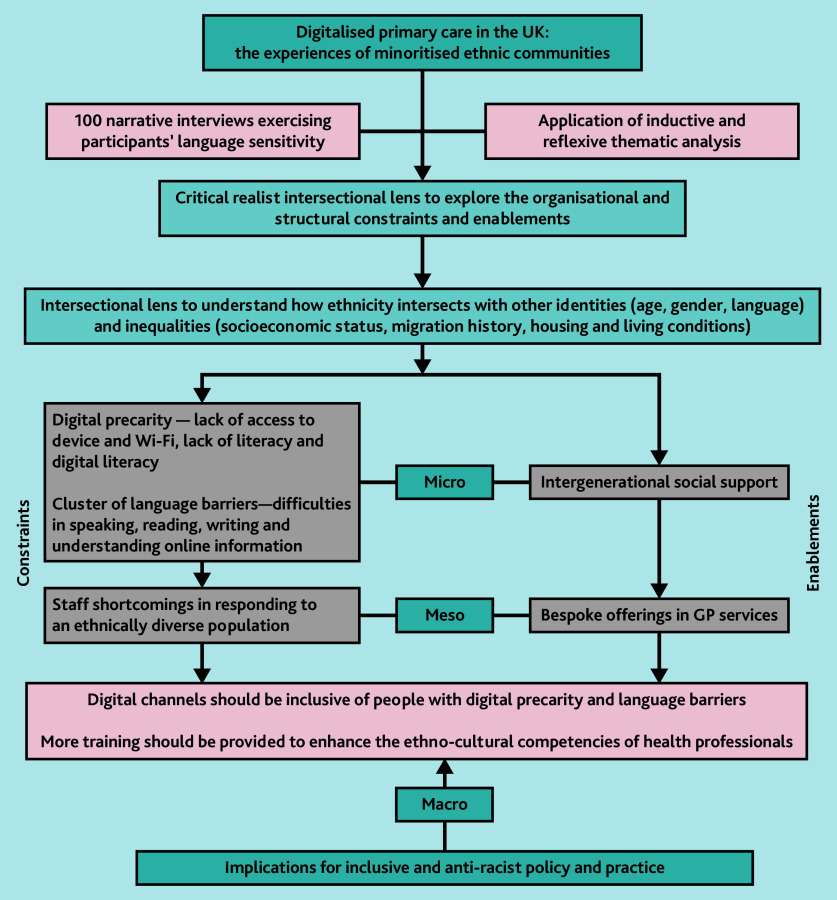
Digitalised primary care in the UK: the experiences of minoritised ethnic communities.

Participant responses indicate that general practices are increasingly directing patients, most of whom are accustomed to using non-digital channels, such as the telephone, to use digital platforms to access services such as appointments and repeat prescriptions. Our intersectional analytical framework enabled us to analyse how inequality, identity, and power relations interact with each other in both the home and the general practice^35^^,^^36^ to facilitate or impede access to digital services. We found that, among many individuals, digital poverty, inadequate digital literacy, a lack of language support, and the absence of other forms of support constrain access to GP services. Some GPs lack the requisite resources and cultural competence to engage with multi-ethnic populations, for instance, in terms of facilitating language support, resulting in delays and exclusion from care. Below we explore the complex ways in which these factors influence the use of digital primary care services. Figure 1 shows the results of our critical realist intersectional approach to qualitative research and the multi-levelled analysis of the enablements and constraints in accessing digital primary care services.

### Clusters of constraints

#### Digital precarity

We have coined the term ‘digital precarity’ to capture the precarious nature of some individuals’ engagement with digital healthcare services owing to a combination of interacting factors including: a lack of access to adequate digital devices and the internet; and limited digital literacy. Disproportionately high levels of poverty among certain ethnic groups, owing to chronic socio-spatial inequality stemming from migratory experiences,^13^^,^^37^^–^41 is associated with many of these factors. Across all age groups, minoritised ethnic individuals on low incomes struggled with the affordability of access to digital primary care as this requires ownership of, or access to, a digital device with sufficient memory space to download GP apps and internet connectivity. Illustrating the way in which ethnicity interacts with low income and housing conditions, individuals living in insecure or temporary housing were sometimes unable to purchase a broadband subscription and hence had to rely on limited mobile data. Such conditional uses of the internet — on a ‘need to use’ basis when accessing health care — is evidenced in the extract below:

*‘When I feel that I need an appointment from doctor then we’ll recharge immediately for one month … I bought the cheapest one for £6 per month. It’s only for calling, and the data is very limited.’*

(Indian Female, 27)

Among those with access to devices, the quality of smartphones also emerged as an access barrier, an issue that appears to be compounded by frequent changes in GP apps. Updating existing and installing new apps takes up space, leaving some individuals with inadequate memory. For instance, a middle-aged Bangladeshi woman reflected:

*‘I downloaded the app* [Dr iQ] *… when you download something, your phone memory becomes full and your phone goes slow …’*

(Bangladeshi Female, 52)

Intersectional analysis revealed that ethnicity interacts with age, migration status, and in some cases gender, to influence access to digitalised health care, as seen in the case of older first-generation immigrants, who either do not have a smartphone or only use it for spoken communication:

*‘Particularly in a South Asian community, it’s really bad … a lot of the older women, they don’t have the phone. They might have a phone just to speak and answer the phone.’*

(Pakistani Female, 65)

The interaction of ethnicity with low income also contributed to digital precarity through limiting opportunities to acquire digital literacy through education and employment, which was compounded by the lack of support available in general practices:

*‘They* [general practice] *do an online service but it’s not user friendly … You need to be a rocket scientist to be able to access that. I’ve tried four times to get it.’*

(Black African Male, 57)

Although enablements in the form of intergenerational social support assist some individuals to access health care, middle-aged women who supported others reported having to share a device to request GP appointments for themselves as well as for their children and older relatives. This was especially evident in the case of female carers who supported large multi-generational Bangladeshi and Pakistani households, including older parents who are not able to use smartphones and do not speak or understand English. As a Pakistani woman, aged 38 years, reflected of her struggle to care for three children and older parents:

*‘Sometimes you’re managing three accounts. Sometimes I’ll be helping my dad, sometimes my mum and the kids accounts as well, so there are a lot of accounts. Especially if your parents don’t speak English or they’re not comfortable with tech.’*

(Pakistani Female, 38)

In situations where non-digital channels were no longer available, some individuals from these communities expressed concern that patients would withdraw from or delay accessing healthcare services owing to the intersecting clusters of constraints that contribute to digital precarity. While some of these challenges may also be experienced by socioeconomically disadvantaged White British individuals, they are likely to disproportionately impact individuals from minoritised ethnic communities owing to the linguistic insensitivity of GPs.

#### Language-related clusters of communication barriers

At the level of general practices, processes of digitalisation appear to have replicated, and in some cases even exacerbated, the lack of linguistic sensitivity of health services already documented in the literature, which is typically manifested in the dominant use of English and the lack of language support for individuals whose first or preferred language is not English.^1^^,^^3^^,^^20^^,^^42^^,^^43^ According to our participants, the vast majority of GP platforms and apps are only available in English.

Intersectional analysis of the ways in which ethnicity interacts with migration status, age, and language use revealed that digitalisation increases the challenges faced by many individuals who are at various stages of acquiring English. For instance, two older Bangladeshi women, who did not have the opportunity to obtain education in either Bangladesh or the UK, as they had migrated as adults, had very limited English language skills. Consequently, they were not able to independently engage with online services at all and typically relied on family members or friends:

*‘I need an interpreter … I can use one of my friends or my next door* [neighbour] *as my interpreter … Without the help of an interpreter, I can’t explain my health issues.’*

(Bangladeshi Female, 70)

While such enablements were sometimes available, these often came at the cost of a loss of privacy.

Others who were able to communicate in spoken English also revealed varying degrees of ability to engage with digital services. Some faced challenges in completing an online consultation form to book an appointment:

*‘If you’re completing an e-consult form you have to write in very descriptive ways … which part of your body is hurting … Otherwise the doctor won’t be able to diagnose … They might not think it’s serious.’*

(Bangladeshi Female, 19)

Securing access to interpreting services for this purpose was extremely difficult, forcing service users to depend on relatives or friends who could assist. This often resulted in delays in accessing timely health care. In some cases, enablements at the level of general practices existed in the form of bilingual staff. However, this was not always available when needed, resulting in delays in accessing timely treatment.

#### Constraints owing to staff shortcomings in responding to an ethnically diverse population

The competence required to diagnose clinical symptoms in an ethnically diverse population, including among individuals with varied skin tones, is crucial to ensuring high-quality healthcare services. However, previous research has revealed the need for continued efforts in this area^43^^,^^44^ as training, educational, and research materials are predominantly modelled on White skin, not only in the UK, but also across the globe. This has resulted in knowledge gaps and incompetencies among GP staff in diagnosing and treating people with non-White skin colours.

We found that some health professionals’ over-reliance on technology, combined with their pre-existing racial stereotypes in assessing disease symptoms through skin colour, could lead to erroneous diagnosis and delayed treatment for people with dark skin. Reinforcing the value of taking an intersectional approach, in this case with regard to the ways in which ethnicity interacts with skin colour in the use of diagnostic tools, some of our interviewees highlighted the problems they encountered when they were asked to send photographs of body parts before being offered an appointment. For example, one of our Black African responders reflected:

*‘Red patches on my skin? … “We can’t see red patches. You haven’t got red patches.” No, because even if I’ve got it, it wouldn’t come across the way it would come across in a normal Caucasian person …’*

(Black African Male, 57)

Some individuals from minoritised ethnic communities expressed concerns regarding digital privacy and data protection, which in some cases deterred individuals from using online GP services. Through intersectional analysis, which considered the ways in which ethnicity and gender interacts with the nature of social networks in areas where certain ethnic groups are concentrated, we found that in Bradford such fears were particularly pronounced and were related in part to prior experiences of data breaches within close-knit communities. Women in particular were concerned about how the health system handles data. Typically, no explanation is provided by GPs on why data are being collected, or with whom they are shared, as well as limits on data sharing. One of our female interviewees in Bradford reflected:

*‘I know of a data breach at my GP. Everybody knows each other and talks around here. There was leakage that happened to my friend, to her extended relatives because of a GP at the practice … I feel nervous about my information … I think that’s what limits me from using online stuff more.’*

(Pakistani Female, 52)

#### The enablements to overcome ethnicity-related constraints

As indicated earlier, a network of support exists for some individuals who are entrapped within the nexus of constraints and at risk of being excluded from digital health services. Our intersectional analysis revealed the interaction of two clusters of enablements: inter-generational social support from individuals who are digitally literate, proficient in English, and available when needed at the household level; and bespoke linguistic support by bilingual receptionists and medical practitioners who are willing to provide interpreting support for booking appointments or participating in virtual consultations at the level of general practices.

#### Intergenerational social support

The existence of intergenerational social support remains prevalent within minoritised ethnic communities with a migration background, especially communities affected by low literacy and language barriers.^45^^,^^46^ Intergenerational social support is not only essential in order to digitally book an appointment, but also is required to describe illness digitally and in person in English with appropriate terminology. One of our middle-aged interviewees of Pakistani heritage reflected:

*‘My mother-in-law, she is about eighty-seven now, not a word of English … she has major health problems … I obviously speak the language and I can hopefully arrange appointments, and stuff like that.’*

(Pakistani Male, 40)

Family and community members help older minoritised ethnic individuals to carry out digital tasks such as filling in online forms and ordering repeat prescriptions. In many cases, those who provide such support do not live in the same household or even the same city. Intergenerational support, in most cases, is accepted and appreciated but often results in exhausting caregiving responsibilities, especially among females. Consequently, some older people were sometimes reluctant to ask for help:

*‘It’s* [GP service] *not accessible … you get your children or your family member to make the appointments or get through them … they* [the older group] *feel uncomfortable, they won’t go out and ask for the support.’*

(Pakistani Female, 65)

Our findings further suggest that some minoritised ethnic individuals who consider themselves to be digitally literate nonetheless require assistance to use online GP services and that such support is not always available when needed. In such cases, access to health care can be delayed, causing distress to care seekers, as evidenced below:

*‘They’re asking for information, and sometimes I’ve struggled because sometimes I don’t know how to upload it* [photos] *to the system … so, I wait for help* [from my son] *and that can delay, and then I miss my appointment.’*

(Pakistani Female, 65)

#### Bespoke offerings in GP services

Encouragingly, our findings also suggest that, in some practices, the presence of bilingual staff enables minoritised ethnic individuals to access services through phone calls and communication with doctors and nurses. This is particularly evident in areas where there is a significant population from a particular ethnic group, such as in Tower Hamlets, where some practices employ Bengali-speaking staff. In Manchester, a number of Chinese interviewees reflected that they preferred to register with a general practice with Chinese-speaking staff even if it was not close to their home. This evidences the importance placed on this type of support by certain minoritised ethnic communities, and the value of an intersectional analysis, which takes into account the interaction of the geographical location of general practices as well as the ethnic composition of the local population.

Further, within the context of the shortcomings of some GPs in responding to an ethnically diverse population and the lack of proactive support in relation to diseases which disproportionately impact certain ethnic groups, it was encouraging to note an initiative in the Manchester area that was facilitated through the internet. To increase access to preventive treatment for prostate cancer among Black African and Caribbean men — who are more vulnerable to the disease than White men^47^ — a specialist clinician made a referral letter available online, enabling men from these ethnic groups to request a priority prostate cancer test.

## Discussion

### Summary

This article explores the multiple ways in which ethnicity interacts with other dimensions of identity and inequality, along with geographical location, within both households and general practices, to mediate access to and engagement with digital primary healthcare services. It is well established that individuals from minoritised ethnic communities have lower levels of satisfaction with respect to access to and use of health services.^3^^,^^4^^,^^6^^,^^20^

The rapid digital transformation of primary care services has presented new challenges to many individuals from such communities.^48^ The main factors underlying these new challenges relate to digital precarity, the lack of linguistic sensitivity of digital infrastructure and health professionals, and the use of diagnostic tools that are not sensitive to differences in skin tone. Further, our interviews suggest that access to digital health services is, to a large degree, enabled by and contingent on the availability of intergenerational support beyond general practices, which places a burden on middle-aged women, in particular to support family members, often with inadequate devices. Further, there are concerns about the risk of data breaches, which are particularly acute in areas where certain minoritised ethnic communities are concentrated.

These unexplored and nuanced insights, combined with pre-existing concerns regarding the risk that digitalisation may increase racialised inequalities, present a compelling argument for greater attention to be paid to engagement with ‘digital first’ primary health care among minoritised ethnic communities. While some examples of good practice have emerged with digital services, overall, little attention appears to have been paid to enabling individuals to overcome the multiple barriers they face. Greater recognition of the differential capacities of individuals from these communities to engage with online services is urgently needed to ensure that racialised inequalities are not replicated or even exacerbated through the digitalisation of primary care.

### Strengths and limitations

While quantitative surveys have been used to evaluate user experiences of primary care services, few efforts have been made to understand such experiences through qualitative exploration.^49^^,^^50^ Since ethno-cultural diversity in the UK has become ‘super-diverse’,^15^ researchers have called for health inequality to be investigated through an intersectional approach, as ethnicity interacts with disadvantaged socioeconomic status in a manner that makes some individuals vulnerable to overlapping forms of discrimination.^1^^,^^19^^,^^51^^–^53 The core strength of this research is that it has delved deeply into minoritised ethnic individuals’ lived experiences through intersectionality-informed narratives, which explore how ethnicity intersects with other dimensions of identity and sociodemographic position to complicate access to online services. Embedded in critical realism, the research has identified the ethnicity-induced factors that intersect with other sociodemographic elements to create constraints or enablements, acting singly or in interaction with each other to facilitate or hinder access to digital health services at both the levels of the household and the general practice.

Limitations of the research include the fact that, owing to our primary focus on access issues related to GP services, we did not explore other avenues of contacting GPs, for example, through out-of-hours services. Further, we have not collected data on how people engaged with online record-keeping systems such as electronic health records (EHR). While such records are widely employed in some geographical areas, their use could lead to further racialised exclusions given the lack of availability of language support to understand and access digital content.

### Comparison with existing literature

Our review of the academic literature on digital exclusion, health inequality, digital health, and access to digital healthcare services revealed that digital exclusion is pronounced among minoritised ethnic communities, a concern that is reinforced by the disproportionate impact of COVID-19 on these communities in the UK.^3^^,^^4^^,^^6^^,^^13^^,^^20^^,^^38^^,^^54^^,^^55^ We also reviewed GP satisfaction surveys,^5^^,^^6^ and UK and Scottish Government documents, including plans and strategies. We found that, while access to health care,^2^^,^^3^^,^^10^^,^^13^ including primary health care,^56^^,^^57^ among minoritised ethnic communities has been extensively discussed, access to digital health care among these communities has received limited attention.

We also examined health inequality-related research and relevant ‘milestone reports’, including: the Black Report,^58^^,^^59^ the Acheson inquiry,^60^^,^^61^ and Marmot’s reviews.^16^^,^^17^ Our analysis indicates that health inequality in high-income countries is intertwined with systemic and racialised disparities*.*^55^^,^^62^*^–^*64 We further found an increasing imperative to understand inequalities in access to and use of healthcare services from an intersectional perspective owing to the limitations of focusing on single dimensions of identity and inequality, and lack of attention to the ethnic composition of patients served by GPs in certain geographical areas. We built on existing scholarship on intersectionality in health services^51^^,^^52^ critical realism,^25^ and feminist organisational studies^21^^,^^35^ to develop a novel theoretical framework that considered the material and social resources available to users within the home and general practices to investigate their engagement with digital primary care services.

### Implications for research and practice

Our application of a critical realist intersectional framework has provided nuanced insights into the multiple ways through which racialised exclusions have been replicated and even exacerbated through processes of digitalisation. It has highlighted the limitations of research that considers only single dimensions of identity or inequality and which is insensitive to the ethnic composition of local populations. Further, the use of this framework has underscored the importance of considering the interaction of factors operating within the home as well as in general practices in considering access to and use of digital services. Urgent attention is now needed to facilitate inclusive and anti-racist policy and practice.

Our findings suggest that ethnicity-induced constraints, such as digital precarity in the form of lack of connectivity to the internet, lack of access to digital devices, and low levels of digital literacy, combined with various levels of proficiency in English, hinder access to health care.^65^^–^67 Such digital exclusions are acute for minoritised ethnic individuals and others on low incomes and with insecure housing tenure who cannot afford digital devices with appropriate bandwidth and mobile data. General practices need to facilitate tailored interventions when recommending apps to patients, taking account of the following issues: the widespread use of smartphones; variation in the quality of such devices; limitations in digital literacy; and the need for more face-to-face support.

Our findings suggest that standardisation in digital services systematically disadvantages some individuals from minoritised ethnic communities, particularly those who are not proficient in English, owing to increased expectations that they communicate illness through digital avenues.^48^^,^^68^ While we have found that certain apps and GP websites offer translation options, some of these are not appropriate to the linguistic needs of the local population, or are not known about by the target populations. Language support should be embedded within ‘digital first’ primary care services to ensure timely access by linguistically diverse populations. For example, at the practice level, facilitating communication in a preferred language, and testing the acceptability of voiceovers, could considerably improve services for those who have some level of proficiency in English and digital literacy. However, such practices need to be driven by national, devolved, and local policies, which prioritise digital equitability.

Ethno-cultural competencies should be included in training health professionals. Lack of knowledge and shortcomings in the cultural competencies of health professionals have hindered minoritised ethnic patients’ access to health care and risk perpetuating or even exacerbating current disparities in health outcomes.^43^^,^^44^ Further, there is a lack of consideration in clinical practice on how assessment and diagnosis can be undertaken for individuals with darker skin colour than the majority White population.^69^^–^71 There is a need for more anti-racist, culturally competent,^65^ and user-friendly access avenues to ensure the effective delivery of ‘digital first’ primary care services. It is hoped that the findings of this study will inform policy and practice with a view to bringing about transformative change.

## Data

To further protect participants’ identity and safeguard sensitive information, the anonymised qualitative dataset will not be made publicly available.
